# Synergistic Effect of Gradient Conductivity and Gradient Microstructures Enabled Ultrasensitive and Ultrabroad Linear Flexible Tactile Sensors

**DOI:** 10.1002/advs.76197

**Published:** 2026-06-23

**Authors:** Yao Fang, Yiwei Wang, Bing Zheng, Liu Yang, Jingyi Yue, Qian Zhou, Jinrong Huang, Yongyun Mao, Qian Li, Jifei Wang, Dongsheng Tang, Yuxin Tang, Bingpu Zhou, Bing Ji

**Affiliations:** ^1^ Key Laboratory of Low Dimensional Quantum Structures and Quantum Control of Ministry of Education School of Physics and Electronics Hunan Normal University Changsha China; ^2^ Hunan Higher Education Key Laboratory of Multiphysics Intelligent Materials and Devices Key Laboratory of Multifunctional Ionic Electronic Materials and Devices Hunan Normal University Changsha China; ^3^ Hunan Research Center of the Basic Discipline for Quantum Effects and Quantum Technologies Hunan Normal University Changsha China; ^4^ School of Physics Central South University Changsha China; ^5^ Yunnan Key Laboratory of Electromagnetic Materials and Devices School of Materials and Energy Yunnan University Kunming China; ^6^ College of Chemical Engineering Fuzhou University Fuzhou China; ^7^ Joint Key Laboratory of the Ministry of Education Institute of Applied Physics and Materials Engineering University of Macau Macau China

**Keywords:** gradient conductivity, gradient microstructures, piezoresistive tactile sensors, ultrahigh sensitivity, ultrawide linear response

## Abstract

The decoupled optimization on sensitivity and linearity is crucial for exploiting high‐performance flexible tactile sensors for diversified applications while remaining challenging. Here, a novel design of conductivity‐microstructures double gradient effect constructed by the rough surface‐based flexible electrode and the arched microstripes‐based carbon‐Polydimethylsiloxane/silver nanowires (CPDMS/AgNWs) electrode is presented. Different from conventional strategies, the top‐down configuration of low‐conductivity CPDMS and high‐conductivity AgNWs enables the pressure‐induced gradient conductivity effect to allow the linearly varied current during the CPDMS deformation. The gradient microstructures of the rough surface and arched microstripes further render the sequential trigger of the gradient conductivity‐induced linear current under different pressures, which accordingly contributes to the ultrawide linearity range upon rationally constructed structural gradient. Besides, the gradient conductivity effect can initially grant the dramatically enhanced sensitivity without depending on structural adjustments. The sensitivity and linearity can thus be optimized without mutual restriction. The proposed sensor exhibits the ultrahigh sensitivity of 5642.02 kPa^−1^ and ultrawide linear response of 0–1560 kPa, which is first reported. The synchronously achieved ultrahigh sensitivity and ultrabroad linearity allow the successful demonstrations of reliable detection of physiological signals for healthcare monitoring, convenient lighting control for smart home, and accurate object identification for intelligent sorting.

## Graphical abstract

A design of double gradient effect is proposed to resolve the contradictory optimization on sensitivity and linearity of piezoresistive tactile sensors. The ultrawide linearity is realized by the sequential trigger of pressure‐induced gradient conductivity‐enabled linear current owing to the gradient microstructures. The ultrahigh sensitivity is endowed by the gradient conductivity effect without depending on structural adjustments, thereby avoiding compromising linearity.

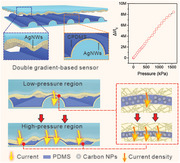

## 1 Introduction

In recent years, flexible tactile sensors that can convert tactile stimuli into detectable electrical signals have attracted remarkable research interests because of their great application potentials for medical healthcare, human‐machine interactions, etc [1, 2, 3, 4, 5]. The reported flexible tactile sensors include piezoresistive, capacitive, piezoelectric, triboelectric and electromagnetic [6, 7, 8, 9, 10, 11]. Comparatively, piezoresistive tactile sensors show more attractiveness with the advantages of simple structure, facile signal acquisition, static and dynamic sensing ability, etc [4, 12, 13]. As sensing devices, the sensitivity and linearity are the most significant parameters. The sensitivity determines the pressure resolution and detection accuracy, whereas the linear sensing capability is critical to maintaining the pressure resolution and simplifying the signal processing [14, 15]. The desired sensors will therefore be demanded with both high sensitivity and broad linearity. The aforetime flexible piezoresistive tactile sensors mainly consist of the solid resistive layer sandwiched between two facing electrodes, and depend on the deformation‐induced resistance variation of the solid resistive layers to reflect the external pressures [16]. Due to the restricted deformability of the solid resistive layer, the sensitivity of these sensors is rather limited. To improve the sensitivity, various conductive materials (e.g., graphene, carbon nanoparticles and nanotubes, metal nanowires, etc.), elastic materials (e.g., Polydimethylsiloxane (PDMS), Ecoflex, Polyurethane, etc.) and microstructures (e.g., micro‐semispheres, micro‐pyramids, porous structures, etc.) have been investigated [17, 18, 19, 20, 21, 22, 23]. The latter has been proved as the most straightforward strategy for sensitivity enhancement because the microstructures can contribute to the dramatically varied contact resistance between the resistive layer and electrode under even low pressures [22, 24, 25]. However, restricted by the stiffening effect, the mechanical deformation of microstructures will be obviously attenuated with increased pressures [15]. The high sensitivity will thus only be effective in low‐pressure regions, resulting in the restricted linearity range of sensors. The commonly employed strategy for linearity improvement is constructing multi‐graded microstructures. Compared with non‐graded microstructures, the involvement of multi‐graded structures can effectively delay the deformation attenuation to a wider pressure region, which accordingly contributes to the obviously increased linearity range [26, 27, 28, 29, 30]. Nevertheless, the delayed microstructural deformation will also restrain the resistance change under equal pressures, leading to the decreased sensitivity. Although the synergistic construction of conductive materials and microstructures has shown the effectiveness in simultaneously optimizing the sensitivity and linearity range, the intrinsic contradiction between ultrahigh sensitivity and ultrabroad linearity is not completely addressed [31, 32, 33]. It is still challenging to the develop the flexible piezoresistive tactile sensors with both ultrahigh sensitivity and ultrabroad linear response range. Besides the sensitivity and linearity, the sensor devices are also desired with low detection limit, fast response/recovery, capability of suppressing or decoupling interferences, and even standalone flexible sensing system for future opportunities [34, 35, 36, 37, 38].

Herein, we presented a novel design of synergistic effect from gradient conductivity and gradient microstructures (i.e., double gradient effect) to address the mutually constrained optimization on sensitivity and linear response of flexible piezoresistive tactile sensors. The gradient conductivity was initially constructed by the top‐down configuration of low‐conductivity (low‐*σ*) carbon‐PDMS (CPDMS) and high‐conductivity (high‐*σ*) AgNWs upon the arched microstripes structure. The gradient structure was configured by associating a rough surface‐based PDMS/AgNWs electrode with the arched microstripes‐based CPDMS/AgNWs electrode. Different from conventional microstructured sensors, the double gradient effect endowed several distinct features to decouple the optimization on sensitivity and linearity. First, the top‐down layout of the low‐*σ* CPDMS and high‐*σ* AgNWs enabled a unique pressure‐induced gradient conductivity, which could allow the linearly varied current during the CPDMS deformation without highly depending on the linear variation in contact area. Second, the gradient structure of the rough surface and the arched microstripes could impose the graded mechanical deformation under pressures, which further allowed the sequential trigger of the gradient conductivity‐induced linear current in low‐pressure and high‐pressure ranges. The ultrabroad linear response could then be guaranteed after linking the linear current within segmented pressure ranges based on the rationally configured structural gradient. Besides, the pressure‐induced gradient conductivity could initially contribute to the dramatically different initial and resultant currents for the significant improvement of sensitivity without depending on structural adjustments. The sensitivity and linearity of sensors could therefore be optimized without mutual restriction. The proposed sensor simultaneously exhibited the ultrahigh sensitivity of 5642.02 kPa^−1^ with the ultrabroad linear response of 0–1560 kPa, which is reported for the first time for piezoresistive tactile sensors. The sensor also exhibited the low detection of 1.8 Pa, fast response/recovery time of below 200 ms, long‐term stability and durability, and excellent anti‐interference capability. The ultrahigh sensitivity and ultrabroad linear response allowed the full range‐available high pressure‐resolution. The sensor could thus be employed to reliably monitor the all‐round physiological signals such as wrist pulse, breathing status, joint flexions and human motions with eliminated influence of wearing tightness. Moreover, the full range‐available high resolution could also allow the sensor to transmit a large number of distinctive signals as coded instructions by simply inputting a series of pressures. The sensor was accordingly demonstrated as a high‐capacity instruction transmitter for lighting control in smart home. In addition, the high resolution of the sensor also allowed the distinguishable increase rate of current signals when grasping objects with different softness or breakage. Assisted by the machine learning, the recognition of softness and breakage for object identification toward smart sorting and quality inspection of intelligent robotics was successfully demonstrated with an average accuracy of 97.25%. With the decoupled optimization on sensitivity and linearity range, it is believed that the novel design of double gradient effect can offer a viable approach to develop high‐performance piezoresistive tactile sensors toward diverse applications.

## 2 Results and Discussion

### 2.1 Design of the Double Gradient Effect

**FIGURE 1:**
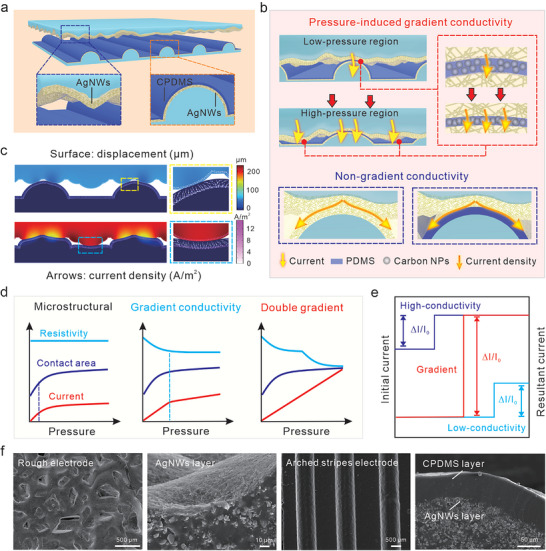
(a) Illustrated composition of the double gradient‐based sensor. (b) Working mechanism of the double gradient effect. (c) Simulation of deformation and current transport of the double gradient‐based sensor. (d) Illustration of the customized linearity range based on the double gradient effect. (e) Principle of the sensitivity enhancement of the gradient conductivity. (f) SEM images of the rough surface‐based AgNWs electrode and the arched microstripes‐based CPDMS/AgNWs electrode.

Figure 1a denotes the illustration of the double gradient‐based piezoresistive tactile sensor. The gradient conductivity was initially constructed by the top‐down layout of low‐*σ* CPDMS layer and high‐*σ* AgNWs upon an arched microstripes array (i.e., striped electrode). The gradient structure was realized by combining a rough surface‐based AgNWs electrode (i.e., rough electrode) with the striped electrode. In this work, the AgNWs layer within the striped electrode and the rough electrode was transferred from the engraving‐fabricated plastic template and the sandpaper via PDMS, respectively. The CPDMS layer within the striped electrode was further obtained by sequentially spraying and curing the CPDMS solution onto the arched microstripes‐based AgNWs layer. The detailed fabrication is presented in the Experimental Section and Figure S1. Figure 1b denotes the working principle of the proposed double gradient effect. Different from conventional strategies which completely rely on the structural regulation for performance optimization, the synergistic effect from the gradient conductivity and gradient structure can endow the decoupled optimization on sensitivity and linearity. Owing to the different conductivities and the top‐down configuration of the low‐*σ* and high‐*σ* layers, the electrons can be imposed to sequentially pierce the low‐*σ* CPDMS layer at the contact position and transport via the high‐*σ* AgNWs layer underneath, as revealed by the simulation results in Figure 1c (see Section S2 for the simulation model establishment). As the CPDMS layer is compressible, the internal carbon nanoparticles (NPs) will be compacted with decreased tunneling resistance accompanied by the reduced thickness of the CPDMS layer. This can induce a decreased resistivity (i.e., increased conductivity) of the CPDMS layer at the contact spot during deformation, which contributes to the gradient conductivity of the CPDMS/AgNWs under pressures (i.e., pressure‐induced gradient conductivity). The associated variation in resistivity and thickness of the CPDMS will derive an additionally varied bulk resistance at the contact sport, thereby relieving the leading role of contact resistance. Moreover, the synergistically varied bulk resistance and contact resistance can further contribute to a linearly increased electrical current without relying on the linear variation in contact area (Figure 1d). In comparison, without the top‐down configuration of low‐*σ* and high‐*σ* components (and hence the pressure‐induced gradient conductivity), the electrons tend to directly transport along the profile of the arched microstripes regardless of the deposited conductive layers (Figure 1b). The entire resistance change will then be always determined by the contact resistance, leading to the high dependence on linear contact area of microstructures for the ensured linearity of sensors (Figure 1d). As a consequence, the gradient conductivity‐based sensors can be endowed with the significantly enhanced linearity range in comparison of the conventional microstructural ones. It should be noted that the linear current variation enabled by gradient conductivity cannot be effective throughout the entire pressure range, because the deformation of low‐*σ* CPDMS will also be restricted by the stiffening effect. After the saturated deformation of CPDMS in high‐pressure range, the gradient conductivity effect will be inactivated, which inevitably yields the attenuated current variation. However, with the gradient structure (and hence the graded mechanical deformation), the microstructure of the rough electrode can contact the bottom of the striped electrode to form an interlock configuration after the obvious deformation of the arched microstripes. This can generate more newly added contact spots to re‐trigger the pressure‐induced gradient conductivity in high‐pressure range, as illustrated in Figure 1b. Such statements can also be confirmed by the simulations results in Figure 1c, where the electrons can sequentially pierce the upper CPDMS layer and transport via the underneath AgNWs layer at the contact spot between the rough electrode and bottom of the striped electrode. As a result, the linear current variation in high‐pressure region can also be guaranteed. From this perspective, the gradient conductivity‐enabled linear current is sequentially triggered by the gradient microstructures under increased pressures, and the overall current variation is actually the linkage of linear current in different pressure regions. By rationally regulating the structural gradient, the change rate of the linear current in high‐pressure region can be maintained consistent with that in low‐pressure region to ensure the ultrawide linearity range of the sensor (Figure 1d). Besides, with the pressure‐induced gradient conductivity, the dramatically different initial and resultant currents of the sensor can be originally endowed, which can accordingly contribute to the ultrahigh sensitivity without depending on structural adjustments (Figure 1e). In this way, it is the synergistic effect of gradient conductivity and gradient microstructures (i.e., double gradient effect) that offers the ultrahigh sensitivity and ultrawide linearity range of the sensor, indeed. Without the gradient microstructures, the gradient conductivity‐enabled linear current will only be effective in low‐pressure regions as mentioned above. On the contrary, without the gradient conductivity, the sensitivity and linearity range will suffer from the mutual contradiction because they both rely on the structural regulation for optimization. With the double gradient effect, the sensitivity and linearity can be separately optimized by the conductive materials and microstructures, respectively. The contradiction between ultrahigh sensitivity and ultrabroad linear response involved in conventional strategies can thus be resolved.

Figure 1d and Figure S2 presents the typical SEM images of the rough electrode and striped electrode of the proposed sensor. The AgNWs was tightly and densely embedded onto the surface of the rough structure and the arched microstripes owing to the transferring operation. The CPDMS layer was fully covered on the AgNWs layer upon the arched microstripe structure (see Figure S3 for the SEM images of the employed AgNWs and carbon NPs). The thickness of the CPDMS layer could be regulated by the employed amount of CPDMS solution in the spraying process (Figure S4). The conductivity of the AgNWs and CPDMS layers could be altered by the AgNWs amount (i.e., layer number) and the carbon content, respectively (Figure S5). Herein, the sheet resistance of the CPDMS layer was constant at ∼3 kΩ cm^−2^ upon a carbon content of 13 wt% for convenient study. Unless specialized statements, the employed AgNWs layer exhibited the sheet resistance of ∼1.3 Ω cm^−2^ upon a layer number of 5, and the CPDMS layer possessed the thickness of ∼45 µm. Reproduced from a 60‐grit sandpaper, the microstructure of the rough electrode possessed a maximum dimension of ∼300 µm and would not change in this work. The arched microstripes exhibited the constant curvature radius of 500 µm while different heights and central spacing, which could be regulated by altering the micro‐carving parameters (see Figure S6 for the detailed characterizations). The optimized arched microstripes possessed the height of ∼350 µm and a central spacing of 2.2 mm.

### 2.2 Double Gradient Effect Construction and Sensing Performance Optimization

**FIGURE 2:**
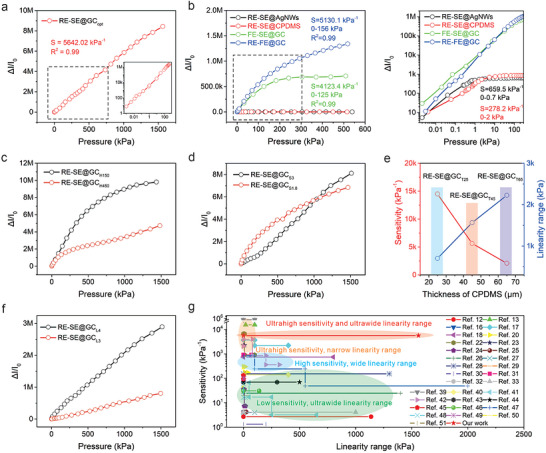
(a) Response curve of the optimized double gradient‐based sensor. (b) Response curves of the sensors with monotonous gradient conductivity and monotonous gradient microstructures. (c,d) Response curves of the double gradient‐based sensors with monotonously changed height (c) and central spacing (d) of the arched microstripes. (e) Linearity range and sensitivity of the double gradient‐based sensors with different CPDMS thicknesses. (f) Response curves of the double gradient‐based sensors with monotonously changed layer number of AgNWs. (g) Comparison of sensing performance among the proposed sensor and other reported literatures.

Figure 2a provides the response curve of the sensor based on the optimized double gradient effect with the mentioned parameters. For convenient statements, the sensor was labelled as RE‐SE@GC_opt_, where “RE” and “SE” represented the rough electrode and striped electrode, respectively, “GC” denoted the gradient conductivity with combined low‐*σ* CPDMS and high‐*σ* AgNWs, and the subscript of “opt” expressed that the employed parameters were optimized. It could be observed that proposed RE‐SE@GC_opt_ sensor exhibited the ultrahigh sensitivity of 5642.02 kPa^−1^ and the ultrabroad linear response of 0–1560 kPa (R^2^ = 0.99). To systematically elucidate the underlying mechanism, the response curve of sensors based on the monotonous gradient structure or gradient conductivity was also examined for comparison, as shown in Figure 2b. Herein, the monotonous gradient structure was constructed by the combined RE and SE fabricated with independent CPDMS (i.e., RE‐SE@CPDMS) or AgNWs (i.e., RE‐SE@AgNWs). The monotonous gradient conductivity was established by associating a flat electrode (FE) with the AgNWs‐based RE or the CPDMS/AgNWs‐based SE. Such a FE was fabricated based on the CPDMS/AgNWs for the AgNWs‐based RE (i.e., RE‐FE‐@GC) and the AgNWs layer for the CPDMS/AgNWs‐based SE (i.e., FE‐SE@GC), respectively. All the parameters were maintained unchanged as mentioned earlier. It was observed that the sensors based on the monotonous gradient structure (i.e., RE‐SE@CPDMS and RE‐SE@AgNWs) exhibited the rather limited sensitivity (maximum of 659.5 kPa^−1^) and linearity (maximum of 0–2 kPa). However, the sensors based on the monotonous gradient conductivity (i.e., RE‐FE@GC and FE‐SE@GC) possessed the much wider linearity range (over 0–120 kPa) and higher sensitivity (5130.1 kPa^−1^ for RE‐FE@GC and 4123.4 kPa^−1^ for FE‐SE@GC). The dramatically enhanced performance could be attributed to the pressure‐induced gradient conductivity effect which relieved the leading role of contact resistance on total resistance variations. Without the gradient conductivity effect, the linearity of sensors was highly relied on the linear variation in contact area of microstructures due to the dominated contact resistance as mentioned above. On the one hand, the stiffening effect would inevitably attenuate the deformation of microstructures under pressures even though the gradient structure was employed, which led to the restricted linearity range of the monotonous gradient structure‐based sensors (Figure 2b). On the other hand, the unchanged conductivity of conductive layers would synchronously affect the initial and resultant current, resulting in the restricted variation of relative current (Figure S7). Therefore, the sensitivity of the monotonous gradient structure‐based sensors was also limited. In comparison, with the pressure‐induced gradient conductivity, an additionally varied bulk resistance at the contact spot could be arisen, which would relieve the leading role of contact resistance. As mentioned above, the different conductivities and top‐down configuration of the low‐*σ* and high‐*σ* components would impose the electrons to sequentially pierce the upper CPDMS and transport via the underneath AgNWs layer. With the CPDMS deformation under pressures, the internal carbon NPs could be compacted with decreased tunneling resistance. The bulk resistivity of the CPDMS layer would thus be decreased along with the thickness reduction, which in turn generated an additional bulk resistance variation at the contact spot (Figure S8). With the synergistic effect of the contact resistance variation, the linearly changed current could then be guaranteed without highly relying on the linear contact area, which therefore contributed to the obviously improved linearity range the GC‐based sensors (i.e., RE‐FE@GC and FE‐SE@GC) in Figure 2b. Such statements could also be proved by the theoretical analysis in Section S3 and the fitting results in Figure S9, where both the RE‐FE@GC and FE‐SE@GC sensors exhibited the linearly varied current in accordance with the experiments. Besides, the pressure‐induced gradient conductivity could also initially guarantee the dramatically different initial and resultant currents (and hence the significantly enhanced relative current variation) without relying on the structural regulation (Figure S7). Therefore, the sensitivity of the GC based sensors was also dramatically enhanced. We also especially examined the response of GC based sensors without microstructures (i.e., flat CPDMS/AgNWs membrane) for comparison (Figure S10). The results indicated that gradient conductivity effect could also ensure a relatively wide linearity range even without microstructures. It should be noted that the gradient conductivity‐induced linear current could not be effective throughout the entire pressure range, because the stiffening effect would also saturate the CPDMS deformation under higher pressures. After the deactivation of pressure‐induced gradient conductivity effect, the attenuated current variations of the monotonous GC based sensors (i.e., RE‐FE@GC and FE‐SE@GC) would still occur in high‐pressure region (Figure 2b). Different from the monotonous GC‐based sensors, the RE‐SE@GC‐based sensors, which associated the gradient structure with the gradient conductivity (i.e., double gradient effect), could be endowed with the customizable current variation in different pressure regions. Owing to the structural gradient, the microstructures of the RE could contact the bottom of the SE to form an interlock configuration in high‐pressure region, as indicated by the optical images capturing the deformation of the RE‐SE@GC_opt_ in Figure S11. This would generate more newly added contact spots to re‐trigger the gradient conductivity effect, which could accordingly guarantee the linearly varied current in high‐pressure region. Note that such a linear current could be independently customized by regulating the gradient structure. Upon the appropriately associated gradient structure, the increase rate of the liner current in high‐pressure range could be maintained consistent with that in low‐pressure range, which therefore contributed to the ultrawide linearity range (0–1560 kPa) of the RE‐SE@GC_opt_ sensor (Figure 2a). Such statements could also be supported by the simulation results in Figure S12. The current density linearly varied with the contact pressure, which was in accordance with the experimental results. It was worthwhile to note that the associated gradient structure did not affect the gradient conductivity‐endowed significant difference of initial and resultant currents (Figure S7; see Figure S13 for the real‐time current variation at the initial stage). Therefore, the RE‐SE@GC_opt_ sensor could also exhibit the ultrahigh sensitivity (5642.02 kPa^−1^) similar as the monotonous GC‐based sensors (Figure 2a,b). Such results revealed that the proposed double gradient effect could address the mutual constrain between ultrahigh sensitivity and ultrabroad linear response involved in conventional strategies.

As discussed above, the appropriate association of the gradient structure is critical to the global linearity of the RE‐SE@GC based sensors. For convenient investigation, we herein regulated the gradient structure association by monotonously altering the height and central spacing of the arched microstripes within the striped electrode (SE) without changing other parameters. To clearly distinguish the specific sensors, the label subscript of RE‐SE@GC would be replaced by the changed parameters. Figure 2c presents the response curves of the RE‐SE@GC‐based sensors with monotonously changed height (i.e., 150 and 450 µm) of the arched microstripes (i.e., RE‐SE@GC_H150_ and RE‐SE@GC_H450_). It could be observed that both the sensors exhibited the obviously decreased linearity range in comparison of the RE‐SE@GC_opt_ with the optimized microstripes height of 350 µm. For the RE‐SE@GC_H450_, the much higher height of the microstripes would delay the contact between the microstructure of RE and the bottom of SE under a much greater pressure, as reflected by the optimal images in Figure S14a. As the microstructures exhibited more serious deformation attention under higher pressures, the re‐triggered linear current in high pressure range would suffer from the relatively lower increase rate, leading to the decreased linearity range (Figure 2c). On the contrary, the much lower height of the microstripes would yield the premature contact (hence the resultantly saturated contact) between the microstructure of the RE and the bottom of the SE under a much lower pressure, as indicated by the optical images capturing the deformation of the RE‐SE@GC_H150_ in Figure S14b. Therefore, the linearity range of the RE‐SE@GC_H150_ was also obviously decreased in comparison of that of the RE‐SE@GC_opt_ (Figure 2a,c). These results sufficiently supported that the height of the arched microstripes within the SE was crucial to the entire linearity of RE‐SE@GC based sensors. In addition, the spacing between adjacent microstripes would also be critical to the entire linearity because it dominated the maximum contact between the RE and SE. The excessively large spacing could impose much more newly added contact spots between the microstructure of the RE and the bottom of the SE in high‐pressure region (Figure S15a). The linear current variation in high‐pressure region would thus be greater than that in low‐pressure region, as indicated by the response curve of the RE‐SE@GC_S3_ with a monotonously increased microstripe central spacing of ∼3 mm in Figure 2d. Conversely, the excessively small spacing would render the limited contact between the microstructure of the RE and the bottom of the SE (Figure S15b). The linearity range would also be restrained similar to that of the monotonous GC‐based sensors, as indicated by the relative current variation of RE‐SE@GC_S1.8_ with a monotonously decreased microstripe central spacing of ∼1.8 mm in Figure 2d. These results sufficiently indicated that the global linearity of the double gradient‐based sensors (i.e., RE‐SE@GC) could be customized by regulating the structural gradient upon the gradient conductivity effect.

It was worth noting that such a customizable linearity was available on the basis of the gradient conductivity effect. As discussed above, the gradient conductivity‐induced linear current is dependent on the deformation of the low‐*σ* CPDMS components. Increasing the thickness of the low‐*σ* components can effectively delay the elastic deformation (and hence the pressure‐induced gradient conductivity effect) to a wider pressure region, which may improve the linear response range of RE‐SE@GC‐based sensors. Such statements could be supported by the further enhanced linearity range (0–2200 kPa) of the RE‐SE@GC_T65_ when monotonously increasing the CPDMS thickness from ∼45 to ∼65 µm, as shown in Figure 2e and Figure S16. On the contrary, the decreased CPDMS thickness would restrict the CPDMS compression within a relatively lower pressure range, which therefore leaded to the reduced linearity range (0–700 kPa) of the RE‐SE@GC_T25_ with monotonously decreased CPDMS thickness of 25 µm. Note that the regulation of CPDMS thickness would also change the resultant bulk resistance (and hence the resultant current), and typically a higher thickness could result in a lower resultant current. The decreased sensitivity of RE‐SE@GC‐based sensors was thus observed when increasing the CPDMS thickness (Figure 2e and Figure S16). Figure 2f further provides the response curves of the RE‐SE@GC_L3_ and RE‐SE@GC_L4_ with monotonously reduced layer number (3 and 4 layers, respectively) of the high‐*σ* AgNWs. Without changing the double gradient configuration, both the sensors exhibited the ultrabroad linear response of 0–1560 kPa same as the proposed RE‐SE@GC_opt_ with the 5‐layered AgNWs. However, the decreased layer number of high‐*σ* AgNWs would reduce the resultant current, thereby causing the decreased sensitivity. These results supported that the sensitivity could be independently optimized by the conductive property of the high‐*σ* components because of the gradient conductivity effect. We also especially compared our proposed sensor with the recently reported sensors, as presented in Figure 2g. To the best of our knowledge, the piezoresistive tactile sensor that possesses such ultrahigh sensitivity (5642.02 kPa^−1^) and ultrabroad linear response range (0–1560 kPa) is first reported [12, 13, 16, 17, 18, 20, 22, 23, 24, 25, 26, 27, 28, 29, 30, 31, 32, 33, 39, 40, 41, 42, 43, 44, 45, 46, 47, 48, 49, 50, 51].

### 2.3 Performance Evaluation of the Proposed Double Gradient‐based Sensor

**FIGURE 3:**
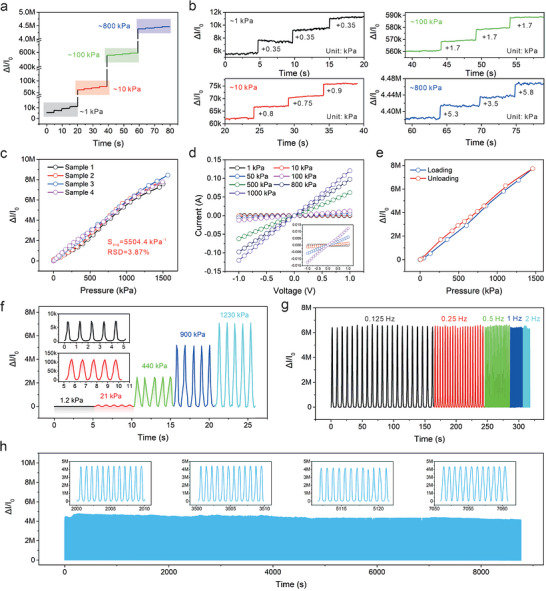
a,b) Overview (a) and enlarged view (b) of the dynamic response to tiny pressure variations upon different pre‐loaded pressures. (c) Relative current variation of the sensor from different samples. (d) I‐V curves of the sensor when applying different pressures. (e) Relative current variation of the sensor during the loading/unloading of pressures. (f) Dynamic response of the sensor under different periodic pressures. (g) Dynamic response of the sensor under different loading frequencies. (h) Dynamic response of the sensor when applying a periodic pressure of ∼800 kPa for over 10 000 cycles.

Figure 3a,b presents the dynamic responses of the sensor to slightly varied pressures upon different pre‐loaded pressures. Attributed to the synchronously obtained ultrahigh sensitivity and ultrabroad linear response, the sensor could exhibit the high resolution throughout the entire pressure spectrum. As a result, the slightly varied pressures (e.g., hundreds to thousands of pascals) could be well distinguished even when different pressures (e.g., 1, 10, 100, and 800 kPa) had been preloaded. Figure 3c shows the relative current variation obtained from different samples of the proposed sensor. All the sensors possessed the ultrahigh sensitivity (5504.4 kPa^−1^ on average) and ultrabroad linear response (0–1560 kPa) with a low value of 3.87% in relative standard deviation (RSD), revealing the excellent reproducibility. Figure 3d presents the I‐V curves of the proposed sensor under pressures ranging from ∼1 to 1000 kPa. The linear relationship under different pressures not only revealed the outstanding electrical stability, but also indicated that the sensor could be easily operated under different voltages. The relative current variation of the sensor during the pressure loading and withdrawing were also examined (Figure 3e). The almost coincident response curves indicated that no obvious hysteresis existed in the process of pressure loading and withdrawing. Figure S17 provides the examined results of detection limit and response/recovery time, where the sensor exhibits a low detection limit of 1.8 Pa and a fast response/recovery time of below 200 ms. The signal drift of the sensor was also evaluated by applying different pressures (Figure S18). The sensor exhibited no obvious signal drift in low‐pressure and medium‐pressure regions (e.g., below 100 kPa). However, the signal drift became relatively apparent under high pressures (e.g., ∼700 kPa), which might be resulted by the creep of elastomers. It should be noted that the current variations induced by the drift was relatively low if the high pressures were not maintained for a long time, which might not significantly affect the sensor performance in real applications. Figure 3f shows the real‐time response of the sensor to different periodic pressures. The signals could be increased with the applied pressure and repeated stably under each of the pressures, revealing the outstanding stability of the sensor. To further evaluate the stability, we also examined the dynamic response of the sensor to the fixed periodic pressure (∼1200 kPa) under different frequencies ranging from 0.125 to 2 Hz (Figure 3g). The signals could be stably repeated with nearly constant intensity, which again supported the outstanding stability of the sensor. The long‐term durability of the sensor was also especially examined by periodically applying the high pressure of ∼800 kPa for over 10 000 cycles (Figure 3h). The waveform of the signals could be well repeated, and the intensity of the signals was not obviously changed after the 10 000‐cycle pressure loading, indicating that the proposed sensor also possessed the outstanding long‐term durability. The long‐term stability of the sensor after days/weeks of storage was also examined by applying a fixed periodic pressure (Figure S19). The results indicated that the response signals could constantly maintain unchanged, which somehow reflected the long‐term electrical stability of the sensor. It should be noted that the employed AgNWs layer is oxidizable. Therefore, the electrical stability of the sensor may be affected if the AgNWs layer is oxidized after storing the sensor for over months/years. However, such a situation may be resolved by replacing the AgNWs with other non‐oxidizable high‐conductivity materials (e.g., AuNWs), because our proposed strategy is not limited to specific conductive materials. Besides, we also evaluated the anti‐interference capability of the sensor by expositing it to the varied environmental conditions (e.g., temperature) and mechanical deformation (e.g., bending). Herein, the sensor was attached onto a hot plate to regulate the temperature. The results indicated that the relative current variations of the sensor were rather tiny even the temperature was significantly varied from 20°C to 70°C, revealing the limited influence of temperature variations on the sensor performance (Figure S20). The relative current variations of the sensor under different bending degrees (e.g., 20°, 30°, 40° and 50°) were also examined, where the sensor was attached onto a PET substrate to quantitatively regulate the bending degree (Figure S21). Different from the environmental conditions, the bending of the sensor could induce additional pressure variations, which therefore led to the relatively larger current variations. However, the value of relative current variations under bending was still very low when compared with that under normal pressures, which also indicated the limited influence of bending deformation on the sensor performance (Figure S21 and Figure 2a). All these results reveal that our sensor possesses the excellent anti‐interference capability, which actually follows the reported decoupling strategy of suppression of interference [34, 37]. We also especially provided a comprehensive comparison table including the sensitivity, linearity range, detection limit, response/recovery time, stability, pressure resolution under high pre‐pressures, footprint, methodology, etc. (Table S1). The comparison results indicates that our proposed sensor also exhibits the comprehensive advantages besides the sensitivity and linearity range.

### 2.4 Demonstrations of Healthcare Monitoring, Intelligent Control and Smart Sorting

**FIGURE 4:**
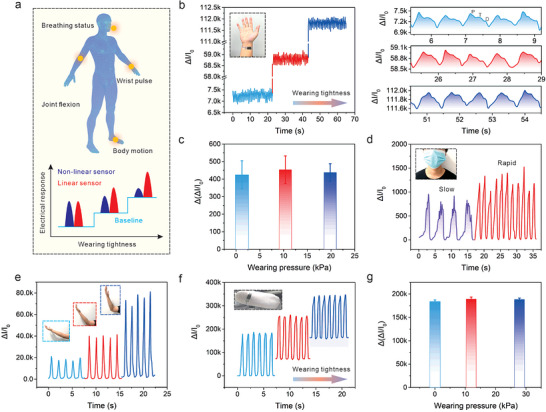
(a) Schematic of all‐round physiological signal monitoring and the influence of wearing tightness. b,c) Dynamic response (b) and corresponding statistical signal intensity (c) of the sensor when detecting artery pulse signals under changed wearing pressures. (d,e) Dynamic response of the sensor to the breathing status (d) and joint flexion (e). (f,g) Dynamic response (f) and corresponding statistical signal intensity (g) of the sensor when detecting walking signals under different wearing pressures.

With the synchronously obtained ultrahigh sensitivity and ultrabroad linear response, the sensor device may be applicable for a wider range of applications. For example, in healthcare monitoring, a single sensor may allow the all‐round detection of pressure‐related physiological signals (Figure 4a). Moreover, the ultrahigh sensitivity and ultrabroad linear response of the sensor may also avoid the influence of wearing conditions for ensured reliability in real applications. As wearable devices, a wearing pressure will be inevitably imposed to the sensor after worn, which may in turn change the real detection region of the sensor. For non‐linear sensors which possess the attenuated resolution with increased pressures, the electrical response to even the identical signals may be dramatically different if the wearing condition is changed, which leads to the questionable detection reliability in real applications (Figure 4a). However, with the synchronously obtained ultrahigh sensitivity and ultrabroad linear response, the sensor can exhibit the maintained resolution throughout the entire pressure spectrum, which can accordingly ensure the constant electrical response to a certain pressure information regardless of the changed wearing conditions. Herein, the dynamic response to the artery pulse was first examined by attaching the sensor onto the wrist of a volunteer with varied tightness, as shown in Figure 4b. In this work, the sensor was attached onto the freely moving human subjects with the assistance of adhesive tapes, as indicated by the inset optical images. The baseline intensity was obviously increased with the enhanced wearing tightness. However, the waveform of the pulse signals with clearly distinguished characteristic peaks of percussion (P), tidal (T) and diastolic (D) could be well repeated. Moreover, the relative pulse intensity maintained nearly constant even the wearing pressure was obviously increased (Figure 4c). Such results indicated that the synchronously realized ultrahigh sensitivity and ultrawide linear response of the sensor could avoid the influence of wearing to guarantee the reliable detection. Figure 4d presents the real‐time response to the breathing status of the volunteer when attaching the sensor onto the face mask. The frequency and intensity of the response signals obviously increased when switching the slow breathing to rapid breathing, indicating the successful recognition of different breathing status. Figure 4e also shows the real‐time response to joint flexions after attaching the sensor onto the elbow joint. The signal intensity was obviously enhanced with the increased flexion degree of the elbow joint. Moreover, the waveform of the response signals could be repeated with almost unchanged intensity under a certain flexion degree, indicating the stable monitoring capability of the sensor. The sensor could also monitor the human motions such as standing, walking and running if attaching the sensor onto the foot, as indicated by Figure S22. The lifting/landing and walking/running actions could be clearly recognized by the obviously varied waveform, frequency and intensity of current signals. Moreover, the signal intensity was almost halved when switching the standing on foot to the standing on feet, which was consistent with the halved plantar pressure, indicating the quantitative detection capability of the sensor in real applications. We also particularly evaluate the reliability of the sensor when detecting the walking with varied wearing tightness (Figure 4f,g). Similar as the response to the wrist pulse, the baseline was obviously increased with the enhanced wearing tightness, whereas the relative signal intensity could be maintained almost unchanged. Such results sufficiently supported that the maintained pressure resolution of the sensor could ensure the detection reliability in real applications with eliminated influence of wearing tightness.

**FIGURE 5:**
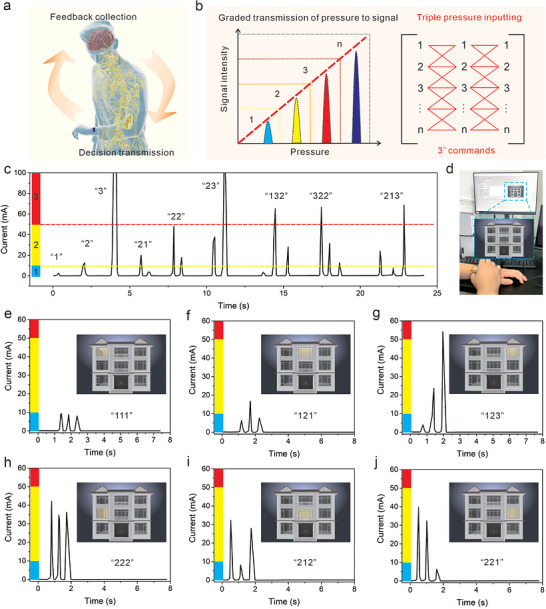
(a) Illustration of the closed loop between feedback collection and decision transmission. (b) Illustration of the multi‐graded pressure inputs and signal outputs for command encoding. (c) Several examples of the 1‐bit, 2‐bit, and 3‐bit signal waveform as encoded commands. (d) Optical image showing the dynamic lighting control for a smart home. (e–j) Different signal waveform as encoded commands for lighting control and optical images capturing the control process.

The reliable detection capability can ensure the direct feedback of targeted information to individual for decision making. Conversely, the resulted decision can also be transmitted to machines if using pressure inputs as the encoded commands. The sensor will thus build a close‐loop between the feedback collection and the decision transmission (Figure 5a). It is worth noting that with the simultaneously obtained ultrahigh sensitivity and ultrawide linear response, the pressure inputs (and the resultant signal outputs) can be easily divided into several non‐overlapping regions to serve as the essential codes of commands (e.g., “1”, “2”, “3”, etc.), as shown in Figure 5b. By inputting the pressures graded in different regions, a large number of encoded commands can then be transmitted by a single sensor for intelligent control applications. For example, if grading the pressure range to *n* levels and employing a triple pressure inputting, up to 3*^n^* different commands can be transmitted by the sensor. Compared with conventional sensors, our proposed sensor does not rely on the multi‐channel array or complicated interface design, which may significantly relieve the device burden for convenient and efficient HMI interfaces [52, 53, 54, 55]. As a proof‐of‐concept, we herein simply graded the pressures into 3 levels (i.e., low pressures of below ∼90 kPa, medium pressures of ∼90–450 kPa, and high pressures of over 450 kPa) as the command codes of “1”, “2” and “3”, respectively. Figure 5c provides several examples of the dynamic signals based on the 3‐code system, including “1”, “2” and “3” via the single pressure inputting, “21”, “22” and “23” via the double pressure inputting, as well as “132”, “322” and “213” via the triple pressure inputting. According to the definition of pressure grades, the codes of “1”, “2” and “3” corresponded to the current signals below 10 mA, within 10–50 mA and over 50 mA, respectively. Note that such wide ranges of current signals (and also pressure inputs) for each of the codes enabled by the ultrawide linearity range and ultrahigh sensitivity of the sensor can dramatically facilitate the operation and signal processing for accurately encoding/transmitting the commands. In one way, each of the codes can be triggered by a wide range of pressure without needing careful loading or specialized training. In another way, the command codes can be recognized more easily and accurately because a large amount of non‐overlapping signals can be ensured for each of the codes. Besides the convenience and accuracy, the capacity of control commands is also a distinct advantage of the sensor. Up to the triple pressure inputting, our sensor could transmit a total of 39 different commands, which could sufficiently satisfy the demand of intelligent control applications (Figure S23). In fact, the capacity of encoded commands could also be significantly improved if further employing multiple pressure inputting. To assess the applicability of the sensor in real applications, we also demonstrated the command transmission based on the triple pressure inputting for controlling the lighting in smart home assisted by LabView. The dynamic signals of the encoded commands (e.g., “111”, “121”, “123”, “222”, “212”, and “221”) and the images capturing the control process are presented in Figure 5d–i. The real‐time control process could also be observed in Video S1. All the results sufficiently revealed the great potentials of the sensor to conveniently serve as a high‐capacity interface for intelligent control applications.

**FIGURE 6:**
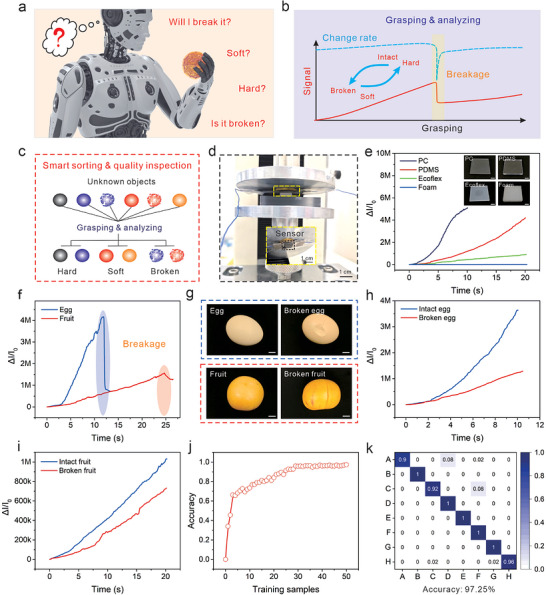
(a) Schematic of the desired grasping functionality of intelligent robotics. (b) The principle of recognizing the softness and breakage of objects during grasping based on the proposed sensor. (c) Illustration of the smart sorting and quality inspection. (d) Optical image of the instrument for imitating the robotic grasping. (e) Response of the sensor when grasping objects with different softness. (f) Response of the sensor when crushing the egg and fruit. (g) Optical images showing the egg and fruit before and after breakage. (h,i) Response of the sensor when grasping an intact/broken egg (h) and fruit (i). (j) Relationship between the accuracy and training samples. (k) Identification results of the broken egg, broken fruit, Ecoflex, egg, foam, fruit, PC, and PDMS (labelled as letters of “A” to “H”).

The full range‐available high resolution of the proposed sensor may also enhance the tactile grasping functionality of intelligent robotics. Beyond the basic grasping, the robotics are also desired with the capability of recognizing the soft/hard features and breakage of unknown objects for intelligent sorting or even quality inspection during grasping (Figure 6a). The judgement of whether the objects are damaged in the grasping process is also expected, which is especially critical to the grasping of fragile products. Such expectations may be realized if attaching the proposed sensor onto the hands of robotics. Typically, the grasping operation will exert compressive strains (and hence the pressures) to both the objects and the sensor, resulting in the continuous increase in current signals during the grasping (Figure 6b). As the softer or broken objects yield lower pressure than the harder or intact ones under equal strain, the increase rate of current signals will be different upon the certain strain rate (i.e., grasping speed). With the full range‐available high resolution of the sensor, the distinct increase rates of current signals when grasping different objects can be well distinguished. The robotics will thus be endowed with the capability of recognizing the soft/hard features and breakage of unknown objects or even the object identification for intelligent sorting and quality inspection (Figure 6c). Moreover, if the objects are broken during grasping, the released pressure will give rise to the dramatic decrease of current signals owing to the high resolution of the sensor (Figure 6b). Such a feature can not only help robotics to judge the dynamic damage of objects during grasping, but also allow the robotics to be trained to avoid scratching fragile products associated with the object identification capability. Herein, the recognition of softness was first demonstrated by clamping objects via a tensile testing machine, where the proposed sensor was attached onto the gripper (Figure 6d). The clamping speed was fixed at a typical value of 3 mm/min to imitate the grasp of robotics. Figure 6e provides the response of the senor to some objects with different soft/hard features, e.g., polycarbonate (PC), PDMS, Ecoflex and foam. It could be observed that the current signals were obviously increased after clamping the objects, and the increase rate was dramatically different owing to the distinct softness. Such distinctive features can further allow the object identification with the assistance of machine learning, which will be discussed in the following. Figure 6f also shows the response of the sensor to some fragile products, e.g., egg and fruit. Similarly, the increased current signals with obviously different increase rates could be observed in virtue of the distinct softness of the egg and fruit. Moreover, the current signals were dramatically decreased even the egg and fruit were slightly broken during the clamping (see Figure 6g for the optical images of the egg and fruit before and after the breakage). Such results indicated the great potential of the sensor to reflect the dynamic breakage of objects during grasping of robotics. The real‐time responses to the intact/broken egg and fruit were also examined for comparison, as shown in Figure 6h,i, respectively. Compared with the intact objects, the broken ones led to the much lower increase rate of current signals, which revealed the potential of the sensor to distinguish the broken objects from intact ones. Note that the sensor with ultrahigh sensitivity and ultrawide linearity range exhibits the advantages of ensuring the high identification accuracy and wide applicability. With the ultrahigh sensitivity, the slight variation in change rate of the current signals can be well distinguished, which therefore enables the accurate recognition of objects with slightly different hardness/softness or even slight breakage. Furthermore, the ultrawide linearity range can allow the sensor to be applicable for a wide range of objects with dramatically varied hardness, which is also especially expected for the dynamic breakage recognition. For example, the pressure that breaks the fragile products such as eggs can reach several hundreds of kilopascals according to the relative current variations during the dynamic breakage and the sensitivity of the sensor (Figures 2a and 6f). For the non‐fragile products, the breakage pressure will be much greater, and the dynamic breakage recognition of such products will inevitably rely on the ultrawide linearity range of the sensor. To further confirm the applicability of object identification, we employed a machine learning model to train and verify the accurate classification of time‐domain signals from the mentioned objects (see Experimental Section for the detailed model establishment). The training result is presented in Figure 6j, where the accuracy of ∼96% could be achieved after training 25 samples. Such a result indicated that the proposed approach did not require massive training datasets to ensure the accuracy. Figure 6k presents the verified results of the broken egg, broken fruit, Ecoflex, egg, foam, fruit, PC, and PDMS (labelled as letters of “A” to “H”). The average accuracy of 97.25% revealed the successful and accurate identification of the objects. The minimum accuracy of 90% was observed for the broken egg, which might be resulted from the deterioration of breakage during the repeated clamping. All these results sufficiently indicated the great potential of the proposed sensor to identify objects during grasping of robotics toward intelligent sorting and even quality inspection.

## 3 Conclusion

A novel design of double gradient effect coordinated by the gradient conductivity and gradient microstructures was proposed to achieve the decoupled optimization on sensitivity and linear response of flexible piezoresistive tactile sensors. The gradient conductivity was initially constructed by the top‐down layout of low‐*σ* CPDMS and high‐*σ* AgNWs upon the arched microstripe array. The gradient structure was configured by associating the rough surface‐based AgNWs electrode with the arched microstripes‐based CPDMS/AgNWs electrode. The different conductivities and top‐down configuration of the low‐*σ* and high‐*σ* components could impose the oriented electrons transport to induce the gradient conductivity effect under pressures, which caused an additionally varied bulk resistance at the contact spot. With the synergistic effect of contact resistance variation, the linearly increased current could then be derived during the CPDMS deformation without highly depending on the linear variation in contact area. The gradient structure of the rough surface and the arched microstripes could further impose the graded mechanical deformation to sequentially trigger the gradient conductivity‐induced linear current in segmented pressure ranges. The ultrawide linear response of the sensor could thus be realized by rationally constructing the structural gradient. Moreover, the pressure‐induced gradient conductivity could originally guarantee the dramatically different initial and resultant currents without depending on the structural regulation. The ultrahigh sensitivity could thus be obtained without affecting the linearity range. The proposed sensor exhibited the ultrahigh sensitivity of 5642.02 kPa^−1^ and ultrabroad linear response of 0–1560 kPa, which is reported for the first time for piezoresistive tactile sensors. The sensor also exhibited the low detection of 1.8 Pa, fast response/recovery time of below 200 ms, long‐term stability and durability, and excellent anti‐interference capability. With the synchronously obtained ultrahigh sensitivity and ultrabroad linear response, the high pressure‐resolution could be maintained throughout the entire pressure spectrum. The sensor could thus be capable of reliably monitoring the all‐round physiological signals, e.g., wrist pulse, breathing status, joint flexions, human motions, etc. with eliminated influence of wearing tightness. Moreover, the full range‐available high resolution enabled the single sensor to output a large amounts of multi‐level signals as encoded commands via pressure inputs. The sensor could thus serve as a high‐capacity smart‐home transmitter for lighting control. Besides, the high performance of the sensor also guaranteed the distinguishable increase rate of current signals during the grasping of objects with different softness and breakage. The recognition of softness and breakage for accurate object identification (with the accuracy of 97.25%) toward smart sorting and quality inspection of intelligent robotic was successfully demonstrated assisted by the machine learning. With the excellent sensing performance and the versatile demonstrations, it is believed that the novel design of double gradient effect can be promising for constructing high‐performance piezoresistive tactile sensors to adapt to diverse application scenarios.

## 4 Experimental Section

### 4.1 Fabrication of the Rough Electrodes:

For the rough surface‐based AgNWs electrode, the AgNWs solution (30–50 µm in length and 100–200 nm in diameter, 20 mg mL^−1^; Nanjing JCNO Technology Co., Ltd, China) with a volume of 100 µL was first dip‐coated onto the surface of a 60‐grit sandpaper. Subsequently, the AgNWs solution with another volume of 100 µL was dip‐spin‐coated (400 rpm, 20s) to obtain the uniform deposition of AgNWs. The mentioned two‐step coating process was repeated 5 times for ensured conductivity of the AgNWs layer. The PDMS‐based gel (Sylgard 184 silicone elastomer kit, Dow Corning, USA) was finally spin‐coated onto the AgNWs‐deposited sandpaper (700 rpm, 20s) with the subsequent curing (80°, 8 h) and peeling‐off operations to obtain the rough electrodes. Herein, the PDMS‐based gel was obtained by uniformly filling the Neodymium‐Iron‐Boron (NdFeB) microparticles (5–10 µm; Magnequench Co., Ltd.) into PDMS for enhanced mechanical properties to facilitate the peeling‐off operation. The detailed fabrication could also be founded in Figure S1. The flat AgNWs electrodes were fabricated with the same process on the plastic board.

### 4.2 Fabrication of the Arched Electrodes

For the arched microstripes‐based electrodes, a plastic template with reversed arched microstripes was first fabricated by the micro‐caving technology with a ball cutter of 1 mm in diameter. The height and central spacing of the arched microstripes could be regulated by changing the micro‐caving parameters. All the arched microstripes were concentrated into an area of 1×1 cm^2^. The two‐step coating of AgNWs solution with the same parameters was employed for the plastic template and repeated certain times for the regulation of the AgNWs layer number. After the same process of spin‐coating of PDMS‐based gel, curing, and peeling‐off, the arched microstripes‐based AgNWs electrodes could be obtained. The CPDMS/AgNWs arched microstripes‐based electrodes were further prepared by sequentially spraying and curing the CPDMS solution on the arched AgNWs electrodes. The detailed fabrication could also be founded in Figure S1. The CPDMS solution was obtained by separately dispersing the carbon nanoparticles (20–50 nm in diameter; Vulcan, China) and PDMS (2 g) into cyclohexane solution (25 mL; Aladdin, China) with certain mass ratios, followed by the uniform mixing of carbon/cyclohexane and PDMS/cyclohexane. During the fabrication of CPDMS solution, the ultrasonic operation (10 min) was employed to guarantee the uniform dispersion and mixing. The sensors were obtained by assembling the rough electrode and arched electrode with facing area of 1×1 cm^2^ via adhesive tapes. Note that the employed approach involved multistep fabrication of transferring the AgNWs, spraying the CPDMS mixture, etc., which might result in the difficulty of fabricating large‐scale high‐density sensor array. However, such an issue may be resolved by employing the in situ printing technologies with appropriate materials, because the proposed strategy of double gradient effect is not limited to specific materials or fabrication methodologies. We will also further optimize the sensor with more facile methodologies for sensor array fabrication in our future work.

### 4.3 Characterizations and Measurements

The optical images were captured by the stereo microscopy (Sanqtid, China). The SEM images were obtained by the scanning electron microscopy (Sigma FE‐SEM, Zeiss Corporation, Germany). The pressures were applied by a motorized motion system (TSA50, Zolix, China) and measured by a high‐precision balance (YP300001D, Lichen, China). The electrical current was recorded by the digital source meter (Keithely 2450, Tektronix Inc., USA) with a typical voltage of 1 V. The sheet resistance and resistivity were measured by a digital multimeter (Keysight 34470, Keysight technologies, USA). The wearable demonstrations were implemented by a volunteer with informed written consent and ensured safety. The ethic approval (2025606) was obtained from the ethics committee at Hunan Normal University prior to the wearable demonstrations.

### 4.4 Machine Learning‐Based Signal Classification

The supervised learning framework based on the random forest algorithm was constructed to achieve accurate classification of time‐domain signals from different objects. The signals from 8‐type target objects were collected under unified experimental conditions to eliminate external interference. The dataset was divided into independent training and test sets by category, with 50 valid samples per category and a total of 800 samples to ensure data balance and integrity. To standardize the input dimension of the model, the 17‐dimensional feature parameters were extracted from each signal, including time features, amplitude statistical features, dynamic variation features, peak features, and energy features. Among these, the amplitude first‐order difference (i.e., instantaneous signal change rate) served as the core discriminative feature. By quantifying the rate of amplitude change between adjacent time points, the characteristic differences of different samples were captured. The statistical parameters, e.g., mean value, standard deviation, maximum value, and minimum value, were extracted to characterize the distribution of the feature, and other auxiliary features supplemented the description of the static statistical properties and the overall energy distribution. The classification model adopted a random forest algorithm consisting of 100 decision trees, with a maximum tree depth of 8 to balance fitting ability and generalization performance. All features were standardized for model training, and 5‐fold cross‐validation was used to verify model stability. The final performance evaluation was conducted on an independent test set to avoid the parameter overfitting and ensure the objectivity of the validation results.

### 4.5 Statistical Analysis

The data were expressed by “mean ± standard deviation (SD)”. The error bars in all data plots denote SD tested from at least four independent samples. All data were performed using Origin software.

## Author Contributions

**Yao Fang**: methodology, data curation, investigation, writing – original draft. **Yiwei Wang**: validation, investigation, methodology, data curation. **Bing Zheng**: methodology, investigation, validation, data curation. **Liu Yang**: methodology, data curation, investigation, validation. **Jingyi Yue**: methodology, validation, data curation. **Qian Zhou**: formal analysis, writing – review and editing, visualization, funding acquisition. **Jinrong Huang**: methodology, investigation. **Yongyun Mao**: formal analysis, writing – review and editing. **Qian Li**: writing – review and editing, funding acquisition. **Jifei Wang**: writing – review and editing, funding acquisition. **Dongsheng Tang**: formal analysis, writing – review and editing. **Yuxin Tang**: formal analysis, writing – review and editing. **Bingpu Zhou**: formal analysis, writing – review and editing. **Bing Ji**: conceptualization, methodology, formal analysis, supervision, funding acquisition, project administration, writing – review and editing, writing – original draft.

## Funding

National Natural Science Foundation of China (No. 62201210, 62401633, 12404475), Natural Science Foundation of Hunan Province (No. 2024JJ6319, 2025JJ20011, 2024JJ5274, 2025JJ60003), Science and Technology Innovation Program of Hunan Province (No. 2025RC3042), Hunan Provincial Major Sci‐Tech Program (No. 2023ZJ1010), Education Department of Hunan Province (No. 23B0074, 23B0066), Hunan province college students research learning and innovative experiment project (No. S202510542269).

## Conflicts of Interest

The authors declare no conflicts of interest.

## Supporting information




**Supporting File 1**: advs76197‐sup‐0001‐SuppMat.docx.

**Supporting File 2**: advs76197‐sup‐0002‐Video S1.mp4.

## Data Availability

The data that support the findings of this study are available from the corresponding author upon reasonable request.
