# Supplementary material for: Synergistic Effect of Gradient Conductivity and Gradient Microstructures Enabled Ultrasensitive and Ultrabroad Linear Flexible Tactile Sensors
Source: Adv Sci (Weinh). 2026 Jun 23;13(51):e76197. doi: 10.1002/advs.76197 (PMC13336898; doi:10.1002/advs.76197)
Supplement: Supplementary file 1 [file ADVS-13-e76197-s002.docx]

Supporting Information

Synergistic Effect of Gradient Conductivity and Gradient Microstructures Enabled Ultrasensitive and Ultrabroad Linear Flexible Tactile Sensors

Yao Fang, Yiwei Wang, Bing Zheng, Liu Yang, Jingyi Yue, Qian Zhou, Jinrong Huang, Yongyun Mao, Qian Li, Jifei Wang, Dongsheng Tang, Yuxin Tang, Bingpu Zhou, and Bing Ji*

Y. Fang, Y. Wang, B. Zheng, L. Yang, J. Yue, J. Huang, Q. Li, J. Wang, D. Tang, B. Ji

Key Laboratory of Low Dimensional Quantum Structures and Quantum Control of Ministry of Education, School of Physics and Electronics, Hunan Normal University, Changsha 410081, China

Hunan Higher Education Key Laboratory of Multiphysics Intelligent Materials and Devices, Key Laboratory of Multifunctional Ionic Electronic Materials and Devices, Hunan Normal University, Changsha 410081, China

Hunan Research Center of the Basic Discipline for Quantum Effects and Quantum Technologies, Hunan Normal University, Changsha 410081, China
E-mail: bingji@hunnu.edu.cn

Q. Zhou

School of Physics, Central South University, Changsha 410083, China

Y. Mao

Yunnan Key Laboratory of Electromagnetic Materials and Devices, School of Materials and Energy, Yunnan University, Kunming 650091, China

Y. Tang

College of Chemical Engineering, Fuzhou University, Fuzhou 350116, China

B. Zhou

Joint Key Laboratory of the Ministry of Education, Institute of Applied Physics and Materials Engineering, University of Macau, Macau 999078, China

**Content**

Section 1. Supplementary figures and table……………………………………………...……3

Section 2. COMSOL model establishment…………………………………………………...15

Section 3. Theoretical analysis on the current variation of the GC-based sensors……………15

Section 4. Supplementary video captions……………………………………………………..19

**Section 1. Supplementary figures**

**Figure S1**. Fabrication of the rough surface-based AgNWs electrodes and the arched micro-stripes-based CPDMS/AgNWs electrodes.

**Figure S2**. a-c) SEM images of the rough surface-based AgNWs electrode (a), the arched micro-stripes-based AgNWs electrode (b), and the arched micro-stripes-based CPDMS/AgNWs electrode (c).

**Figure S3**. a-c) SEM images of the employed AgNWs (a), carbon black nanoparticles (b) and NdFeB microparticles (c).

**Figure S4**. Thickness regulation of the CPDMS layer via the spraying volume of the CPDMS solution.

**Figure S5**. a) Sheet resistance of the CPDMS layer with different contents of carbon NPs in PDMS. b) Sheet resistance of the AgNWs layer with different layer numbers.

**Figure S6**. a) Optical images of the arched micro-stripes-based electrode with the stripe height of ~150 μm, ~350 μm and ~450 μm. b) Optical images of the arched micro-stripes-based electrode with the stripe central spacing of ~3 mm, ~2.2 mm and ~1.8 mm.

**Figure S7**. Initial current without loading pressures, resultant current under a pressure of ~500 kPa and the corresponding relative current variation of different sensors.

**Figure S8**. Normalized variation in resistivity of the CPDMS layer with the underneath AgNWs film under pressures. The resistivity was measured by a flat CPDMS/AgNWs membrane with a PET/ITO electrode to avoid the influence of contact resistance.

**Figure S9**. a-b) Theoretical fitting results of the FE-SE@GC (a) and RE-FE@GC based sensors (b) with the monotonous gradient conductivity effect.

**Figure S10**. Relative current variation of the GC based sensor without microstructures. Herein, the GC based sensor without microstructures was assembled by the flat CPDMS/AgNWs membrane and the ITO/PET electrode for convenient examination.

**Figure S11**. Optical images capturing the dynamic deformation of the RE-SE@GC_opt_.

**Figure S12**. Simulation results of the variation in normalized current density with the displacement and contact pressure.

**Figure S13**. Real-time current of the RE-SE@GC_opt_ sensor before and after applying pressures at the initial stage.

**Figure S14**. a-b) Optical images capturing the dynamic deformation of the RE-SE@GC_H450_ (a) and RE-SE@GC_H150_ (b) with different heights of the arched micro-stripes.

**Figure S15**. a-b) Optical images capturing the dynamic deformation of the RE-SE@GC_S3_ (a) and RE-SE@GC_S1.8_ (b) with different stripe central spacings.

**Figure S16**. Relative current variation of the RE-SE@GC_T25_, RE-SE@GC_T45_ and RE-SE@GC_T65_ with different thicknesses of the CPDMS layer.

**Figure S17**. a-b) Examination results of the detection limit (a) and the response/recovery time (b) of the sensor.

**Figure S18**. Real-time current signals of the sensor when maintaining different pressures.

**Figure S19**. Real-time response of the sensor to the fixed periodic pressure after storing the sensor over days/weeks.

**Figure S20**. Relative current variations of the sensor under different temperatures.

**Figure S21**. Relative current variations of the sensor under different bending degrees.

**Figure S22**. a-b) Real-time response of the sensor to the standing (a), waling and running (b).

**Figure S23**. a) Encoded commands triggered by the single and double pressure inputting. b-d) Encoded commands triggered by the triple pressure inputting beginning with “1” (b), “2” (c) and “3” (d).

**Table S1**. Comparison among our work and other reported literatures regarding sensitivity, linearity range, detection limit, response/recovery time, stability, pressure resolution under large pre-pressures, footprint, methodologies, etc.

| **Sensitivity (kPa⁻¹)** | **Linearity Range (kPa)** | **Limit of Detection (Pa)** | **Response/Recovery Time (ms)** | **Stability (Cycles)** | **Pressure Resolution under Large Pre-pressure** | **Footprint (Size / Thickness).** | **Methodology** | **Ref.** |
| --- | --- | --- | --- | --- | --- | --- | --- | --- |
| 2.69 | 0-1138 | 5.67 | 13.77 / 18.73 | 2,000 (at 5 kPa) | ~916 Pa under 14.37 kPa | 1×1 cm² / - | sandpaper casting, solution casting | 1 |
| 15525 | 20-100 | - | 30 / 31 | 3,000 (at 50 kPa) | - | - / - | magnetic alignment, laser engraving | 2 |
| 21.67 | 0-43 | 3.4 | 60 / 30 | 5,000 (at 5 kPa) | - | - / - | laser marking, in-situ polymerization | 3 |
| 3656.8 | 0-100 | 1.1 | 6/12 | 10,000 (at 200 kPa) | - | ~1 cm² / - | spray coating | 4 |
| 742.3 | 0-800 | - | 40 / 30 | 10,000 (at 210 kPa) | - | ~5×5 mm² / - | laser reduction, transfer printing | 5 |
| 298.4 | 1.4-15.7 | 7.1 | 7/16 | 10,000 (at 7.14 kPa) | - | - / - | electrospinning, screen printing | 6 |
| 6667.21 915 | 0-10 10-100 | 1.3 | 18 / 30 | 15,000 (at 8 kPa) | 200 g under 1300 kPa | 1×1 cm² / - | photolithography, plasma enhanced chemical vapor deposition | 7 |
| 2235.84 | 0-10 | ~12 | ~120 / - | 1,000 (at 25 kPa) | - | - / - | salt template, dip-coating | 8 |
| 35.02 | 0-8 | - | 6 / - | 1,400 | - | - / 200 µm | sandpaper molding | 9 |
| 4.2 | 0-25 | 0.5 | 150 / 150 | >2000 (at 5 kPa) | - | - / - | photolithography, wet etching | 10 |
| 24.6 | 0-1400 | - | - / - | 50,000 (at 130 kPa) | - | 9x9 mm²/ - | infrared picosecond laser | 11 |
| 3.997 | 0-100 | 4.7 | 120 / 60 | >5000 (at 100 kPa) | - | 21×58.5 mm² / 0.158 mm | screen printing | 12 |
| 153.3 | 0.0005-1300 | 0.5 | 40 / 30 | 20,000 | 0.1 kPa under 255 kPa | 1×1 cm² / - | sandpaper molding | 13 |
| 20.25 | 0-35 | - | <400 / - | ~5,000 | - | 10×10 mm² / - | direct ink writing | 14 |
| 1.3 | 0-200 | 35 | 12.5 / 37.5 | >10,000 | - | 1×1 cm² / - | 3D printing | 15 |
| 924.37 | 0-70 | 0.83 | - / - | >22,000(at 100 kPa) | - | 2×2 cm² / - | spin coating, spray coating | 16 |
| 380000 | 0-100 | 0.025 | 0.016 / - | 8,000 (at 80 kPa) | 0.1 kPa under 50 kPa | - / - | photolithography | 17 |
| 4.11 | 0-3800 | ~12 | ~100 / 50 | >1200 (at 3000 kPa) | ~20 kPa under up to 3000 kPa | ~200 µm | solution casting, screen printing | 18 |
| 400000 | 0-100 | - | 30 / 20 | ~10,000 | - | - / ~200 µm | femtosecond laser | 19 |
| 138.6 | 0-400 | 3 | 34 / 39 | 24,000 (at 70 kPa) | 70 Pa under 100 kPa | - / - | infrared laser processing | 20 |
| 67.1 | 0-650 | 7 | 40 / 37 | 10,000(at 127.4 kPa) | - | - / - | 3D printing | 21 |
| 2071.8 3987 860 362.5 | 0-0.6 0.6-80 80-200 200-320 | ~11 | 0.4 / 2.4 | 53,000 (at 50 kPa) | 150 Pa under 250 kPa | - / - | screen printing, spray coating | 22 |
| 69.8 | 0-300 | - | 1/4 | 5,000 (at 300 kPa) | - | - / 180 µm | sand paper template casting | 23 |
| 1.7 | 0.002-500 | 2 | 63 / 47 | 6,000 (at 30 kPa) | - | - / - | 3D printing | 24 |
| 93.17  132.02 | 0-68.37 68.37-148 | 0.0133 | 20 / 20 | 400 (at 90% pressing strain) | - | 1×1×1 cm³ | solution dipping | 25 |
| 30.8 | 0-140 | 0.15 | 64 / 68 | 5,500 (at 300 Pa) | 50 Pa under 100 kPa | 1×1 cm² / - | natural loofah fabric | 26 |
| 839.1 | 0-100 | 1.3 | <80 / <80 | 10,000 (at 500 kPa) | - | - / - | bio-templating, 3D printing | 27 |
| 59.7  16.76 | 0-0.1 0.1-15 | 0.186 | 72 / 96 | 20,000 (at 50% strain) | - | 1.1×1.1 cm² / - | freeze drying, carbonization | 28 |
| 348.7 | 0-4 | 0.92 | 12/14 | - | - | - / 2.4 mm | chemical synthesis, spin-coating | 29 |
| 5.7 0.5 | 0-8 8-15 | - | - / - | 200 (at 5 kPa) | - | - / 3 mm | self-assembly | 30 |
| 0.0351 0.00372 | 0-20 20-1500 | 80 | ~600 / - | 1000 (at 2 kPa) | - | - / - | in-situ polymerization | 31 |
| **5642.02** | **0-1560** | **1.8** | **<200 / <200** | **>10,000 (at ~800 kPa)** | **<3.5 kPa at 800 kPa** | **1×1 cm² /**  **<1 mm** | **micro-carving, spray coating** | **This work** |

**Section 2. COMSOL model establishment**

The simulation model was established via the COMSOL Multiphysics software. The geometric structure of the arched micro-stripes-based electrode was built with the similar dimension in accordance with the experiments (i.e., 500 μm in radius of curvature and 350 μm in height of the arched microstripes, 45 μm in thickness of the CPDMS layer, 20 μm in thickness of the AgNWs layer, etc.). The geometric structure of the rough electrode was simply established with the combination of a larger arc-shaped body (350 μm in radius of curvature and 200 μm in height) and two smaller arc-shaped bodies (250 μm in radius of curvature and 100 μm in height) for convenient simulation. The detailed geometric structure can be referred to Figure 1c. The PDMS with the redefined elastic modulus of 3 MPa and other parameters default was set as the elastomer. The default conductivity of carbon and Ag was employed for the CPDMS and AgNWs for convenient simulation, respectively. The solid mechanics and electric current interface were utilized to trace the mechanical deformation and current density with the governing equations of $\nabla\cdot{FS}^{T}+F_{v}=0$ and $\nabla\cdot J=Q$. In the equation, $F_{v}$ is the volume force vector. $S=C : \varepsilon$, where $C$ is a parameter related to the elastic modulus $E$ and Piosson’s ratio $\nu$, and $\varepsilon=\frac{1}{2}\left[ ({\nabla u)}^{T}+\nabla u+({\nabla u)}^{T}\nabla u \right]$ is a parameter related to the displacement vector $u$. $J=\sigma(-\nabla V)+J_{e}$, where $\sigma$ is the conductivity, $V$ is the potential and $J_{e}$ is the external current density. The contact conditions were set as friction and adhesion. The contact impedance was set as 0.003 $\Omega\cdot m^{2}$ for the interface between the CPDMS layer and the AgNWs layer of the arched micro-stripes-based electrode. The electrical contact was set for the contact interface between the two electrodes. The mesh was controlled with the maximum size of 20 μm at the contact interface. The voltage input for the current density traction was set as 1 V. The prescribed displacement constraint was employed to facilitate the mechanical simulation.

**Section 3. Theoretical analysis on the current variation of the GC-based sensors**

For GC-based sensors, the total resistance variation is dominated by both the additionally varied bulk resistance of the CPDMS layer and the contact resistance between the electrodes. According to the definition of bulk resistance ($R_{b}$) and contact resistance ($R_{c}$), we will have

$R=R_{b}+R_{c}=\frac{\rho_{b}t_{b}}{A}+\frac{\rho_{c}}{A}$	(S1)

where $\rho_{b}$ and $t_{b}$ are the bulk resistivity and thickness of the CPDMS at the contact spot, respectively, $\rho_{c}$ is the contact resistivity, and $A$ is the contact area between the electrodes.^[32]^ We have discussed that the CPDMS deformation at the contact spot will condense the embedded carbon NPs to reduce the internal tunneling resistance, which therefore contributes to the decreased bulk resistivity. Assuming that the tunneling resistivity of carbon NPs is $\rho_{t}$, the internal tunneling resistance can be expressed by

$R_{t}=\frac{\rho_{t}}{A_{t}}$	(S2)

where $A_{t}$ is the contact area between adjacent carbon NPs.^[32]^ According to Hertz contact theory, such a contact area can be expressed by

$A_{t}=\pi\left[ \frac{3D_{t}(1-{\nu_{t}}^{2})}{E_{t}} \right]^{\frac{2}{3}}\cdot F^{\frac{2}{3}}$	(S3)

where $D_{t}$, $\nu_{t}$ and $E_{t}$ are the diameter, Posson’s ratio and elastic modulus of the carbon NPs, respectively.^[33]^ As the bulk resistivity variation is originated from the internal tunneling resistance, we will have

$\rho_{b}\propto R_{t}=\frac{\rho_{t}}{A_{t}}=\frac{\rho_{t}}{\pi\left[ \frac{3D_{t}(1-{\nu_{t}}^{2})}{E_{t}} \right]^{\frac{2}{3}}}\cdot F^{-\frac{2}{3}}$	(S4)

Given that $\rho_{t}$, $D_{t}$, $\nu_{t}$ and $E_{t}$ are all constants for certain carbon NPs, and the pressure is resulted from the applied force, Equation S4 can be simply expressed by

$\rho_{b}\propto P^{-\frac{2}{3}}$	(S5)

Such a relationship can also be supported by the experimental results in **Figure S8**. Besides the bulk resistivity, the thickness variation of the CPDMS at the contact spot will also affect the bulk resistance. According to the definition of elastic modulus, the thickness of the CPDMS layer at the contact spot can be written as

$t_{b}=t_{b0}(1-\frac{F}{AE})$	(S6)

where $t_{b0}$ and $E$ are the initial thickness and elastic modulus of the CPDMS layer, respectively. Note that the bulk resistivity of the CPDMS layer at the contact spot will also determine the contact resistivity (i.e., $\rho_{c}\propto\rho_{b}$) because of the direct contact between the upper electrode and the CPDMS layer. The total resistance variation can then be rewritten as

$R=\frac{\frac{\rho_{t}}{\pi\left[ \frac{3D_{t}(1-{\nu_{t}}^{2})}{E_{t}} \right]^{\frac{2}{3}}}\cdot F^{-\frac{2}{3}}\cdot t_{b0}(1-\frac{F}{AE})}{A}+\frac{\frac{\rho_{t}}{\pi\left[ \frac{3D_{t}(1-{\nu_{t}}^{2})}{E_{t}} \right]^{\frac{2}{3}}}\cdot F^{-\frac{2}{3}}}{A}$	(S7)

after combining Equation S1, S4 and S6.

For FE-SE@GC based sensor, the contact area between the flat electrode and the arched micro-stripes can be expressed by

$A=2\sqrt{\frac{D(1-\nu^{2})}{\pi LE^{*}}}\cdot F^{\frac{1}{2}}$	(S8)

where $D$ and $L$ are the diameter of curvature and length of the arched micro-stripes, respectively, $E^{*}$ is the elastic modulus of the flat electrode and the arched micro-stripes, and $\nu$ is the corresponding Posson’s ratio.^[34]^ By combining Equation S7 and S8, the total resistance variation of the sensor can then be written as

$R=\left( \frac{\rho_{t}t_{b0}}{\gamma\alpha}+\frac{\rho_{t}}{\gamma\alpha} \right)F^{-\frac{7}{6}}-\frac{\rho_{t}t_{b0}}{\gamma\alpha^{2}E}F^{-\frac{2}{3}}$	(S9)

where $\gamma=\pi\left[ \frac{3D_{t}(1-{\nu_{t}}^{2})}{E_{t}} \right]^{\frac{2}{3}}$ and $\alpha=4\sqrt{\frac{DL(1-\nu^{2})}{\pi E^{*}}}$. Under a certain voltage of 1V, the electrical current of the FE-SE@GC based sensor will thus be

$I=\frac{1}{\frac{\rho_{t}}{\gamma\alpha}\left[ {(t}_{b0}+1)F^{-\frac{7}{6}}-\frac{t_{b0}}{\alpha E}F^{-\frac{2}{3}} \right]}\propto\frac{1}{\left[ {(t}_{b0}+1)F^{-\frac{7}{6}}-\frac{t_{b0}}{\alpha E}F^{-\frac{2}{3}} \right]}$	(S10)

The fitting result can then be obtained by setting the typical value of the constant parameters in Equation S10. In this work, the arched micro-stripes possessed the diameter of curvature of 1 mm and length of 1 cm, and the CPDMS layer exhibited the initial thickness of 45 μm. For convenient calculation, the elastic modulus of the CPDMS and the elastomer for the flat electrode and the arched micro-stripes was typically set as 2 and 3 MP, respectively, and the Posson’s ratio was set as 0.49. The pressure can be calculated by the applied force and the floor area of the micro-stripe. The fitting result indicated that the FE-SE@GC based sensor exhibited the linear current variation in accordance with the experiments, as shown in **Figure S9**.

For the RE-FE@GC based sensor, the microstructure with different dimensions of the rough electrode will sequentially contact the flat electrode under pressures, which accordingly contributes to the sequential variation in bulk resistance and contact resistance at different contact spots. To simplify the analysis, we herein assume that the microstructure of the rough electrode is an array of two combined micro-domes with different heights (150 and 250 μm) according to previously reported literatures.^[35]^ The contact area between a flat surface and a micro-dome can be expressed by

$A=\pi\left[ \frac{3D(1-\nu^{2})}{8E} \right]^{\frac{2}{3}}\cdot F^{\frac{2}{3}}$	(S11)

according to Hertz contact theory, where $D$, $E$ and $\nu$ are the diameter, elastic modulus and Posson’s ratio of the micro-dome.^[33]^ Combining Equation S7 and S11, the total resistance variation will thus be

$R=\frac{\rho_{t}}{\gamma\beta}F^{-\frac{4}{3}}\left[ t_{b0}\left( 1-\frac{F^{1/3}}{\beta E} \right)+1 \right]$	(S12)

where $\gamma=\pi\left[ \frac{3D_{t}(1-{\nu_{t}}^{2})}{E_{t}} \right]^{\frac{2}{3}}$ and $\beta=\pi\left[ \frac{3D(1-\nu^{2})}{8E} \right]^{\frac{2}{3}}$. Under a certain voltage of 1V, the electrical current will be

$I=\frac{F^{\frac{4}{3}}}{\frac{\rho_{t}}{\gamma\beta}\left[ t_{b0}\left( 1-\frac{F^{1/3}}{\beta E} \right)+1 \right]}$	(S13)

Such an expression can be used to represent the current variation when the taller micro-domes contact the flat electrode. Similarly, the electric current variation when the shorter micro-domes contact the flat electrode will be

$I^{'}=\frac{{F^{'}}^{\frac{4}{3}}}{\frac{\rho_{t}}{\gamma\beta}\left[ t_{b0}\left( 1-\frac{{F^{'}}^{1/3}}{\beta E} \right)+1 \right]}$	(S14)

where $F^{'}$ is the force applied to the shorter micro-domes. As the conductive paths when both the taller and shorter micro-domes contact the flat electrode are connected in parallel, the total current will thus be

$I_{total}=\frac{F^{\frac{4}{3}}}{\frac{\rho_{t}}{\gamma\beta}\left[ t_{b0}\left( 1-\frac{F^{1/3}}{\beta E} \right)+1 \right]}+\frac{{F^{'}}^{\frac{4}{3}}}{\frac{\rho_{t}}{\gamma\beta}\left[ t_{b0}\left( 1-\frac{{F^{'}}^{1/3}}{\beta E} \right)+1 \right]}\propto\frac{F^{\frac{4}{3}}}{\left[ t_{b0}\left( 1-\frac{F^{1/3}}{\beta E} \right)+1 \right]}+\frac{{F^{'}}^{\frac{4}{3}}}{\left[ t_{b0}\left( 1-\frac{{F^{'}}^{1/3}}{\beta E} \right)+1 \right]}$	(S15)

Note that $I^{'}$ will only be effective when the excess part of the taller micro-domes has been deformed. According to Hertz contact theory, the deformation distance (i.e., height variation) can be expressed by^[33]^

$d=\left[ \frac{{9(1-\nu^{2})}^{2}}{{8DE}^{2}} \right]^{\frac{1}{3}}F^{\frac{2}{3}}$	(S16)

The current $I^{'}$ will thus be taken into account when $d=100 \mu m$ according to the assumption of the height of micro-domes (150 and 250 $\mu m$). Moreover, when both the taller and shorter micro-domes contact the flat electrode, they will exhibit the equal deformation distance, which can be used for the assigned value of $F$ and $F^{'}$. The fitting results can thus be obtained after setting the value of constant parameters in Equation S15. Herein, the diameter of the micro-domes was typically set as 1 mm, and the elastic modulus and the Posson’s ratio were also set as 3 MPa and 0.49, respectively. The pressure was calculated by the total force and the floor area of the two combined micro-domes. The floor area of the two combined micro-domes (i.e., the microstructure unit) was roughly set as 1.25 mm^2^ according to the SEM images and the previously reported literatures.^[35]^ The fitting result indicated that the RE-FE@GC based sensor also exhibited the liner current variation in accordance with the experiments, as shown in **Figure S9**.

**Section 4. Supplementary video captions**

**Video S1**. The real-time control of lighting in smart home.

**References**

1.	Z. Xiong, J. Huang, J. Chen, et al., “Synergy of Hierarchical Structures and Multiple Conduction Mechanisms for Designing Ultra-Wide Linear Range Pressure Sensors,” *Chemical Engineering Journal* 503, (2025): 158359.

2.	Y. Jiang, F. Liang, H. Y. Li, et al., “A Flexible and Ultra-Highly Sensitive Tactile Sensor through a Parallel Circuit by a Magnetic Aligned Conductive Composite,” *ACS Nano* 16, (2022): 746-754.

3.	S. Wang, Y. Yao, W. Deng, et al., “Mass-Produced Skin-Inspired Piezoresistive Sensing Array with Interlocking Interface for Object Recognition,” *ACS Nano* 18, (2024): 11183-11192.

4.	C. Zhang, S. Lang, M. Tao, et al., “Deep Learning-Assisted Piezoresistive Pressure Sensors with Broad-Range Ultrasensitivity for Wearable Motion Monitoring,” *Nano Energy* 140, (2025): 2100859.

5.	F. Luo, A. Ciesielski, and P. Samorì, “Nonlinear Conductive Graphene Composites for Pressure Sensing with a Linear Response and Voltage‐Driven Thermal Correction,” *Advanced Materials* 37, (2025): 2503867.

6.	M. Chao, L. He, M. Gong, et al., “Breathable Ti3c2tx Mxene/Protein Nanocomposites for Ultrasensitive Medical Pressure Sensor with Degradability in Solvents,” *ACS Nano* 15, (2021): 9746-9458.

7.	P. Li, G. Tai, W. Luo, et al., “Skin-Inspired Graded Micro-Conformal Tunneling Interface for Piezoresistive Sensors with Broad-Range Ultra-Sensitivity,” *Chemical Engineering Journal* 503, (2025): 158470.

8.	R. Luo, Y. Cui, H. Li, et al., “Fragmented Graphene Aerogel/Polydimethylsiloxane Sponges for Wearable Piezoresistive Pressure Sensors,” *ACS Applied Nano Materials* 6, (2023): 7065-7076.

9.	T. Liu, G.-y. Gou, F. Gao, et al., “Multichannel Flexible Pulse Perception Array for Intelligent Disease Diagnosis System,” *ACS Nano* 17, (2023): 5673-5685.

10.	S. W. Kim, J.-H. Lee, H. J. Ko, et al., “Mechanically Robust and Linearly Sensitive Soft Piezoresistive Pressure Sensor for a Wearable Human–Robot Interaction System,” *ACS Nano* 18, (2024): 3151-3160.

11.	R. Chen, T. Luo, J. Wang, et al., “Nonlinearity Synergy: An Elegant Strategy for Realizing High-Sensitivity and Wide-Linear-Range Pressure Sensing,” *Nature Communications* 14, (2023): 6641.

12.	X. Wang, G. Wu, X. Zhang, et al., “Traditional Chinese Medicine (Tcm)‐Inspired Fully Printed Soft Pressure Sensor Array with Self‐Adaptive Pressurization for Highly Reliable Individualized Long‐Term Pulse Diagnostics,” *Advanced Materials* 37, (2024): 2410312.

13.	J. Chen, K. Chen, J. Jin, et al., “Outstanding Synergy of Sensitivity and Linear Range Enabled by Multigradient Architectures,” *Nano Letters* 23, (2023): 11958-11967.

14.	M. Zhu, C. Chen, A. Yu, et al., “Multilayer Step-Like Microstructured Flexible Pressure Sensing System Integrated with Patterned Electrochromic Display for Visual Detection,” *ACS Nano* 19, (2025): 19488-19496.

15.	Z. Xu, D. Wu, Z. Chen, et al., “A Flexible Pressure Sensor with Highly Customizable Sensitivity and Linearity Via Positive Design of Microhierarchical Structures with a Hyperelastic Model,” *Microsystems & Nanoengineering* 9, (2023): 5.

16.	B. Ji, Q. Zhou, J. Wu, et al., “Synergistic Optimization toward the Sensitivity and Linearity of Flexible Pressure Sensor Via Double Conductive Layer and Porous Microdome Array,” *ACS Applied Materials & Interfaces* 12, (2020): 31021-31035.

17.	Y. Lee, J. Myoung, S. Cho, et al., “Bioinspired Gradient Conductivity and Stiffness for Ultrasensitive Electronic Skins,” *ACS Nano* 15, (2020): 1795-1804.

18.	X. Lin, Y. Teng, H. Xue, et al., “Janus Conductive Mechanism: An Innovative Strategy Enabling Ultra‐Wide Linearity Range Pressure Sensing for Multi‐Scenario Applications,” *Advanced Functional Materials* 34, (2024): 2316314.

19.	M. Zeng, J. Ding, Y. Tian, et al., “Phase Separation Manipulated Gradient Conductivity for a High‐Precision Flexible Pressure Sensor,” *Advanced Functional Materials* 34, (2024): 2411390.

20.	R. Chen, C. F. Cheung, Q. Zhang, et al., “Ultra‐Sensitive and Linear Flexible Pressure Sensors with Tri‐Scale Graded Microstructures for Advanced Health Monitoring and Robotic Perception,” *Advanced Science* (2025): e16810.

21.	S. Tu, Y. Xi, X. Cui, et al., “Skin-Inspired Interlocked Microstructures with Soft-Hard Synergistic Effect for High-Sensitivity and Wide-Linear-Range Pressure Sensing,” *Chemical Engineering Journal* 496, (2024): 154083.

22.	Z. Han, L. Mo, S. Han, et al., “Flexible Sensors with Enhanced Sensitivity and Broadened Detection Range through Conformal Printing and Space‐Confined Design,” *Small* 21, (2024): 2407168.

23.	N. Bai, D. Xu, Z. Su, et al., “Dual‐Graded Microstructure Engineering for Flexible Piezoresistive Sensors with High Sensitivity and Broad Linear Range in Physiological Monitoring,” *Advanced Science* 12, (2025): e07135.

24.	Y. Li, Y. Wang, X. Wang, et al., “Linear Range Enhancement in Flexible Piezoresistive Sensors Enabled by Double‐Layer Corrugated Structure,” *Advanced Functional Materials* (2025): e13480.

25.	X. Xu, and B. Yan, “Bioinspired Luminescent Hof‐Based Foam as Ultrafast and Ultrasensitive Pressure and Acoustic Bimodal Sensor for Human–Machine Interactive Object and Information Recognition,” *Advanced Materials* 35, (2023): 2303410.

26.	C. Lu, Y. Shen, X. Chan, et al., “Loofah Fiber Fabric‐Based Piezoresistive Pressure Sensor with Wide‐Linear‐Range High Sensitivity,” *Small* 21, (2025): e06521.

27.	Z. Lu, P. Liao, J. Song, et al., “Pushing Boundaries: Nature-Inspired Hybrid Microstructured Flexible Sensors with Enhanced Detection Range,” *Chemical Engineering Journal* 521, (2025): 166839.

28.	M. Jiang, B. Li, Y. Zhao, et al., “Superelastic Biomass‐Based Carbon Aerogels Reinforced by Vein‐Like Networks and Pillar Structures for Piezoresistive Sensing, High‐Temperature Applications, and Supercapacitors,” *Advanced Functional Materials* (2025): e13514.

29.	F. u. Nisa, M. Tahir, S. Khalid, et al., “Supramolecular Cross-Linking Enables Highly Stretchable and Ultrasensitive Polyurethane-Poly(3,4-Ethylenedioxythiophene) Tactile Sensors,” *ACS Nano* 19, (2025): 35102-35118.

30.	Y. Wang, Q. Geng, H. Lyu, et al., “Bioinspired Flexible Hydrogelation with Programmable Properties for Tactile Sensing,” *Advanced Materials* 36, (2024): 2401678.

31.	G. Chen, Y. Zhang, S. Li, et al., “Flexible Artificial Tactility with Excellent Robustness and Temperature Tolerance Based on Organohydrogel Sensor Array for Robot Motion Detection and Object Shape Recognition,” *Advanced Materials* 36, (2024): 2408193.

32.	G. R. Ruschau, S. Yoshikawa, R. E. Newnham, “Resistivities of conductive composites”, *Journal of Applied Physics* 72, (1992): 953.

33.	E. Dintwa, E. Tijskens, H. Ramon, “On the accuracy of the Hertz model to describe the normal contact of soft elastic spheres”, *Granular Matter* 10, (2008): 209.

34.	Q. J. Wang, D. Zhu 2013. “Chapter H-Hertz Theory: Contact of Cylindrical Surfaces.” in Encyclopedia of Tribology. First Ed., edited by Q. J. Wang, Y.-W. Chung, Heidelberg: Springer. ISBN 978-0-387-92896-8.

35.	X. Tang, C. Wu, L. Gan, e. al., “Multilevel Microstructured Flexible Pressure Sensors with Ultrahigh Sensitivity and Ultrawide Pressure Range for Versatile Electronic Skins”, *Small* 15, (2019): 1804559
